# Knowledge, attitudes, and beliefs about acute coronary syndrome among patients with type 2 diabetes*

**DOI:** 10.1590/1518-8345.5435.3503

**Published:** 2021-11-19

**Authors:** Camille Alardis Hunte Johnson, Natássia Condilo Pitta, Carina Aparecida Marosti Dessotte, Rosana Aparecida Spadoti Dantas, Lídia Aparecida Rossi

**Affiliations:** 1 Charles Roza School of Nursing, Linden, Guyana.; 2 Universidade de São Paulo, Escola de Enfermagem de Ribeirão Preto, PAHO/WHO Collaborating Centre for Nursing Research Development, Ribeirão Preto, SP, Brazil.

**Keywords:** Knowledge, Diabetes, Acute Coronary Syndrome, Attitudes, Beliefs, Nursing, Conocimiento, Diabetes, Síndrome Coronario Agudo, Actitudes, Creencias, Enfermería, Conhecimento, Diabetes, Síndrome Coronariana Aguda, Atitudes, Crenças, Enfermagem

## Abstract

**Objective:**

to evaluate the knowledge, attitudes, and beliefs of Guyanese individuals with type 2 diabetes regarding acute coronary syndrome and explore associations between these measures and the population’s sociodemographic and clinical characteristics.

**Method:**

cross-sectional study conducted in Linden, Guyana, with sixty type 2 diabetics, interviewed using a sociodemographic and clinical questionnaire and the Acute Coronary Syndrome-Response Index. The Mann-Whitney test was used to assess potential differences between groups according to the ACS-Response Index subscales, and sex, age, time since diabetes diagnosis, and body mass index and the Kruskal-Wallis test to compare the ACS-Response Index subscales according to educational level.

**Results:**

only two participants correctly answered more than 70% of the Knowledge subscale. Participants obtained low mean scores in all subscales. Less than half of the participants reported chest pain and arm pain as symptoms of heart attack. Significant differences were found when comparing Knowledge (*p*=0.008) and Attitudes (*p*=0.009) according to educational level.

**Conclusion:**

individuals with type 2 diabetes showed low level of Knowledge, Attitudes, and Beliefs. Participants who scored the highest in Knowledge and Attitudes presented the highest educational level. The results show a need for health professionals to heed knowledge deficits regarding acute coronary syndrome among type 2 diabetes.

## Abstract (es)

**Objetivo:**

evaluar el conocimiento, actitudes y creencias de guayaneses con diabetes tipo 2 sobre el síndrome coronario agudo y explorar la asociación entre esas mediciones y las características sociodemográficas y clínicas de esa población.

**Método:**

estudio transversal realizado en Linden, Guayana, con sesenta diabéticos tipo 2 entrevistados usando un cuestionario sociodemográfico y clínico y el *Acute Coronary Syndrome-ACS Response Index*. El test Mann-Whitney fue usado para verificar posibles diferencias entre grupos de acuerdo con las subescalas del *ACS-Response Index* y sexo, edad, tiempo de diagnóstico, e índice de masa corporal, y el test Kruskal-Wallis para comparar las subescalas del *ACS-Response Index* y el nivel de escolaridad.

**Resultados:**

dos participantes respondieron correctamente más de 70% de las preguntas de la subescala Conocimiento. Los participantes obtuvieron bajas puntuaciones en todas las escalas. Menos de la mitad respondieron que el dolor en el pecho y brazo son síntomas de ataque cardíaco. Diferencias significativas fueron encontradas entre el nivel de Conocimiento (*p*=0.008) y Actitudes (*p*=0.009) y de escolaridad.

**Conclusión:**

diabéticos tipo 2 presentaron bajo nivel de Conocimiento, Actitudes y Creencias. Los participantes con puntuaciones más altas en las subescalas conocimientos y actitudes tuvieron nivel educacional más alto. Los resultados mostraron que profesionales de salud deben prestar atención al conocimiento de los diabéticos tipo 2 sobre el síndrome coronario agudo.

## Abstract (pt)

**Objetivo:**

avaliar o conhecimento, atitudes e crenças de guianenses com diabetes mellitus tipo 2 sobre a síndrome coronariana aguda e explorar a associação entre essas medidas e características sociodemográficas e clínicas desta população.

**Método:**

estudo transversal realizado em Linden, Guiana. Sessenta pacientes com diabetes tipo 2 foram entrevistados usando um questionário sociodemográfico e clínico e o *Acute Coronary Syndrome-Response Index*. O teste Mann-Whitney foi usado para verificar possíveis diferenças entre grupos de acordo com sub-escalas do *ACS-Response Index* e sexo, idade, tempo de diagnóstico, e índice de massa corporal, e o teste Kruskal-Wallis para comparar as sub-escalas do *ACS-Response Index* e escolaridade.

**Resultados:**

dois participantes responderam corretamente mais de 70% das questões da sub-escala Conhecimento. Os participantes obtiveram baixas pontuações em todas as sub-escalas. Menos da metade respondeu que dor no peito e no braço são sintomas de ataque cardíaco. Diferenças significativas foram encontradas entre os níveis de Conhecimento (*p*=0.008) e Atitudes (*p*=0.009) e escolaridade.

**Conclusão:**

participantes com diabetes mellitus tipo 2 apresentaram níveis baixos de Conhecimento, Atitudes e Crenças. Participantes com pontuações mais altas no Conhecimento e Atitudes apresentaram nível educacional mais alto. Os resultados mostram que profissionais da saúde precisam atentar para déficits de conhecimento de pacientes com diabetes tipo 2 sobre a síndrome coronariana aguda.

## Introduction

Non-communicable diseases, such as cardiovascular diseases, cancer, diabetes, and chronic respiratory diseases, are the leading cause of death and disability worldwide^(1)^. Diabetes, for example, is a high-risk factor for cardiovascular diseases^(2)^. In 2017, the estimated prevalence of diabetes among individuals aged 18 to 99 was 451 million worldwide, projected to be 693 million by 2045^(3)^. Moreover, lower and middle-income countries have the most significant health burden of non-communicable diseases, as over three-quarters of deaths from these conditions occur in these countries^(4)^.

In 2016, cardiovascular diseases were the leading cause of death in Guyana, accounting for 34% of deaths among 30 to 69 year-old-individuals^(5)^. In addition, a recent study^(6)^ addressing the national diabetes prevalence in the country found a rate of 18.1% among adults, with a higher prevalence in women (21.4%) than men (15.1%).

Due to a lack of knowledge, diabetic individuals often fail to engage in self-care activities to control and prevent cardiovascular diseases, such as coronary artery disease. Additionally, acute coronary syndrome (ACS) is frequently asymptomatic in diabetic patients, leading to a delay in seeking assistance^(7)^ and contributing to inappropriate self-care behaviors. Low levels of knowledge and inability to interpret and respond appropriately to ACS symptoms are problems faced globally^(8)^.

The knowledge, attitudes, and beliefs of individuals diagnosed with ischemic heart disease are associated with delays in seeking help^(9)^. Proper knowledge may motivate people to take care of their health^(10)^ and inspire them to respond appropriately in case of a heart attack^(11)^. However, knowledge about ACS and its symptoms is insufficient to persuade a person to seek the needed early treatment^(12-13)^. Therefore, it is important to consider the psychosocial factors involved, such as personal beliefs, values, and emotions^(12)^, and the context where people live.

Guyana is currently facing a challenge in its healthcare system, which places little emphasis on preventing ACS among patients with diabetes. The education of patients with type 2 diabetes is mainly focused on preventing complications, such as peripheral vascular disease, hypertension, and stroke. Furthermore, there is no evidence of the level of knowledge among people with diabetes on acute coronary syndrome; individuals with diabetes are at high risk for this condition. Such knowledge can offer a basis for educational interventions suitable for this population to improve self-efficacy in the self-management of cardiovascular complications, which are among the most common causes of mortality among Guyanese citizens^(4)^. Thus, it is essential to assess the knowledge and beliefs of patients with type 2 diabetes on ACS and their attitudes when responding to its symptoms.

The knowledge, attitudes, and beliefs of type 2 diabetic patients toward ACS were assessed in this study using the Acute Coronary Syndrome Response Index (ACS-Response Index). The ACS-Response Index was developed, based on Leventhal’s Self-regulatory Model of Illness Behavior^(9)^, to assess cognitive and emotional aspects related to the patients’ response in the face of a heart attack. However, Leventhal’s Common-Sense Model of Self-regulation explains the management of chronic conditions in everyday life^(14)^. According to this Model, a patient’s behaviors and feelings about the disease’s symptoms are based on physical experiences. These experiences allow patients to identify symptoms and form their beliefs about them. Thus, the patients’ perspectives about the illness and its treatment are constructed^(14)^. This index was developed and validated to assess patients’ knowledge, attitudes, and beliefs toward coronary heart disease and their response to its symptoms^(9)^. From this perspective, responses to a health threat such as ACS are associated with a patient’s ability to perceive and locate symptoms, revealing one’s relationship with the disease. Thus, knowledge, attitudes, and beliefs influence a patient’s perceptions about the ACS^(9)^.

Previous studies have addressed the knowledge, attitudes, beliefs, and perceived risks of ACS and its symptoms among patients diagnosed with ischemic heart disease^(8,13,15-17)^. However, no studies addressing the knowledge, attitudes, and beliefs regarding ACS among type 2 diabetic patients were found, which is the focus of this study. Recently, researchers performed a scoping review to analyze 16 articles addressing the knowledge and perceptions of patients with type 2 diabetes towards cardiovascular disease prevention, reporting that patients with type 2 diabetes could not identify the factors associated with cardiovascular disease. As a result, they had a knowledge deficit related to the disease at the time of diagnosis, failing to recognize cardiovascular risk factors and manage them. In addition, knowledge and perceptions were associated with the patients’ demographic characteristics such as years of instruction, race, age, and region of residence^(18)^.

Deficits in knowledge, lack of financial and social support, in addition to beliefs and attitudes, were identified as factors influencing self-care among Chinese individuals with acute coronary syndrome and diabetes^(19)^. The ACS-Response Index was used to assess knowledge, attitudes, and beliefs toward the ACS symptoms among 50 Lebanese patients after a myocardial infarct: 12 (24%) of whom had diabetes^(15)^. The author found a lack of knowledge concerning heart attack symptoms and inconsistent beliefs about the need to seek help in the event of a heart attack and perceptions concerning their ability to control their disease^(15)^.

No studies were found on the knowledge, attitudes, and beliefs of Guyanese patients with type 2 diabetes about ACS symptoms. Identifying the perceptions of this group toward ACS symptoms is paramount and contributes to planning and improving educational programs focused on the ability of patients to recognize symptoms and respond to a heart attack appropriately and timely. Therefore, this study aims to evaluate the knowledge, attitudes, and beliefs of Guyanese individuals with type 2 diabetes about acute coronary syndrome and explore associations between these measures and this population’s sociodemographic and clinical characteristics.

## Method

### Study design and participants

This is a cross-sectional study, reported according to the Strengthening the Reporting of Observational Studies in Epidemiology (STROBE) statement. Data were collected from June 2019 to January 2020, at the Linden Hospital Complex, in Region 10, Guyana. The Linden Hospital Complex includes three hospitals (two district hospitals and one regional hospital). The study participants were selected from the Mackenzie Hospital (the regional hospital) and the Upper Demerara Regional Hospital (one of the district hospitals). The Mackenzie Hospital has 115 beds and provides general medicine, general surgeries, obstetrical and gynecologist, pediatric, and neonatal care, while the Upper Demerara Regional Hospital provides outpatient services, such as antenatal, chronic disease, and family health clinics. The availability of resources constrained the period of data collection. The participants were selected by consecutive and non-probabilistic sampling identified through the hospital database by consulting hospitalization charts and follow-up visit schedules at the outpatient clinic.

Inclusion criteria were adult patients with type 2 diabetes, regardless of sex, with cognitive competence to answer the questionnaire. Participants were considered cognitively competent if they answered questions concerning their current home address, current date, and time. Additionally, patients diagnosed 36 months ago or later were included. The reason is that metabolic abnormality atherogenic dyslipidemia occurs before diabetes is clinically diagnosed; thus, early education regarding ACS is vital for these patients^(20)^. Exclusion criteria were persons residing in difficult-to-access communities without public transportation and individuals not attending the follow-up during the data collection period.

### Measures

#### Sociodemographic and Clinical data Questionnaire

A questionnaire was developed to collect sociodemographic and clinical data, including date of the interview, date of birth, sex, marital status, level of education [primary, secondary, trade (postsecondary) and university level], time since the diabetes diagnosis, weight and height, physical activities (walking, running and riding - bike), and comorbidities.

#### Acute Coronary Syndrome Response Index (ACS-Response Index)

The ACS-Response Index was developed to assess the knowledge, attitudes, and beliefs concerning ACS symptoms and responses to symptoms^(9)^. It consists of 33 questions divided into three subscales, which measure Knowledge, Attitudes, and Beliefs. With 21 dichotomous items, the subscale Knowledge addresses various symptoms related to ACS (15 questions) and unrelated to ACS (six questions - reverse code). One point is assigned to each correct answer and zero points to each incorrect answer. This subscale’s total score is obtained by adding correct answers, ranging from 0 to 21. The subscale Attitudes contains five items (22 to 26), and the subscale Beliefs contains seven items (27 to 33). These subscales include questions about seeking help in an emergency and the respondents’ perceptions regarding self-confidence in recognizing symptoms. These two subscales include a four-point ordinal scale, ranging from one to four. The Attitudes subscale includes the following possible answers: not at all=1, little sure=2, pretty sure=3, and very sure=4. The possible answers for the Beliefs subscale are strongly agree=1, agree=2, disagree=3, and strongly disagree=4. The Beliefs subscale has three reverse coded items (27, 31, and 33). The total score ranges from 5 to 20 for the Attitudes subscale and 7 to 28 for the Beliefs subscale. Higher scores in any of the subscales reflect more positive responses to the ACS symptoms. This instrument should be used as an index rather than a scale. Therefore, as an index that assesses observed facts, the objective is to compare the scores of each subscale (Knowledge, Attitudes, and Beliefs) separately as concepts that influence responses regarding ACS^(9)^.

Dr. Barbara Riegel authorized the use of the ACS-Response Index. The internal consistency obtained in the validation study of the original English version was 0.82 for the Knowledge subscale and 0.76 for both the Attitudes and Beliefs subscales^(9)^. English is the primary language spoken in Guyana; however, considering cultural differences between the population in which the instrument was tested and the population addressed in this study, the ACS-Response Index was tested on ten patients from three health facilities to ensure the instrument’s content and face validity. These ten patients were selected according to the same inclusion and exclusion criteria previously mentioned. All the patients answered the items without making any queries, and there was no need to adjust the instrument. Therefore, these ten participants were not included in the final sample. The instrument’s reliability was also tested in this study’s final sample. The internal consistency of the Knowledge subscale assessed by the Kuder-Richardson-20 coefficient (KR-20) was 0.962, given this subscale’s dichotomous answer format. Cronbach alpha coefficients obtained for the Attitudes and Beliefs subscales were 0.830 and 0.798, respectively. Values equal to or higher than 0.65 for the KR-20 and 0.70 for the Cronbach’s alpha were considered acceptable^(21)^.

### Data collection

The first author selected potential participants from the medical records in the Mackenzie Hospital and the Upper Demerara Regional Hospital. The potential participants were contacted via telephone and at the medical clinics. Eight out of 84 eligible patients no longer resided at the addresses found on their charts; six did not respond to the invitation to participate in the study; five refused to participate; two emigrated to other countries, and three worked outside Linden. Thus, the sample consisted of 60 participants. The hospital database was accessed manually to collect the patients’ sociodemographic and clinical data. Data not available in the medical records were collected through interviews. Data collection was conducted by the first author through face-to-face interviews, according to the availability of participants and in the places indicated by them.

### Data analysis

Data were processed and analyzed using IBM SPSS Statistics for Windows, Version 24.0 (Armonk, NY: IBM Corp.), and descriptive analyses were performed for all the variables.

The Mann-Whitney test was performed to assess potential differences between groups according to the scores obtained in the ACS-Response Index subscales and sex (male *versus* female), age (younger than 60 years old *versus* 60 years old or older), time since diabetes diagnosis (less than 12 months or between 12 and 36 months) and body mass index (not overweight) and overweight (overweight and obesity grade I, II and III)^(22)^. In addition, the Kruskal-Wallis test was used to compare the scores obtained in the ACS-Response Index subscales and educational levels [primary, secondary, or trade (postsecondary - technical)/university]. A p-value less than or equal to 0.05 was considered statistically significant in all the tests.

### Ethical consideration

The Research Ethics Committee (IRB) approved the proposal in March 2019 (No. 508/2019). All the participants signed free and informed consent forms after receiving clarification about the study’s objectives and procedures; participation was voluntary.

## Results

Of the 60 (100%) individuals who participated in the study, predominantly women (65%). Ages ranged from 27.9 to 89.8 years old, and the mean age was 55 (Standard Deviation - S.D.=11.6). Most participants had completed the secondary level of education. The mean time since participants were diagnosed with type 2 diabetes was 23.2 months. Hypertension was the main comorbidity reported (70%). The majority of the participants had some level of obesity, and 76.7% were engaged in some form of exercise activity (Table 1).

**Table 1:** Sociodemographic and clinical characteristics of the 60 participants. Linden, Guyana, 2020

Variable	*N* (%)
*Sex*	
Female	39 (65)
Male	21 (35)
Age (years) Mean=55; S.D.*=11.6	
*Educational level*	
Primary	25 (41.7)
Secondary	26 (43.3)
University	6 (10.0)
Trade School	3 (5.0)
*Time since diabetes diagnosis (months) Mean=23.2; S.D. = 9.5*	
*Comorbidities (yes)*	54 (90.0)
Hypertension	42 (70.0)
Coronary Artery Disease	4 (6.7)
Neuropathy	10 (16.7)
Nephropathy	3 (5.0)
Retinopathy	20 (33.3)
*Body mass index (yes) Mean= 31.4; S.D. = 5.7* **eutrophic**	
Eutrophic	6 (10.0)
Overweight	19 (31.7)
Obesity I	19 (31.7)
Obesity II	14 (23.3)
Obesity III	2 (3.3)
*Physical activity (yes)*	46 (76.7)
Walking	38 (63.3)
Running	4 (6.7)
Riding	3 (5.0)

* S.D. = Standard Deviation

The number of correct answers provided to each item of the Knowledge subscale and the percentage of correct answers was calculated for each participant (Table 2). Only two participants (3.3%) correctly answered more than 70% of the questions in the Knowledge subscale. The total mean score obtained in this subscale was 9.12. Less than half of the participants were able to identify common symptoms of a heart attack, such as chest pain/pressure/tightness (44.1%), arm pain (45%), and sweating (30%).

**Table 2:** Descriptive statistics for the Knowledge subscale items - Acute Coronary Syndrome - Response Index (ACS-Response Index). Linden, Guyana, 2020

ACS*-Response Index Knowledge subscale	Total mean score (S.D.)^†^ 9.12 (3.4)
Correct answer for each item	Total median score (range) 9.0 (4-16)
	*N* (%)
1. Lower abdominal pain^‡^ (N=59)	43 (72.9)
2. Arm pain or shoulder pain (N=60)	27 (45.0)
3. Arm paralysis^‡^ (n=56)	43 (76.8)
4. Back pain (N=58)	25 (43.1)
5. Chest pain/pressure/tightness (N=58)	26 (44.1)
6. Chest discomfort (heaviness, burning, tenderness) (N=60)	23 (38.3)
7. Cough^‡^ (N=56)	40 (71.4)
8. Dizziness, light headedness (N=57)	19 (33.3)
9. Headache^‡^ (N=59)	31 (52.5)
10. Heartburn/indigestion/stomach problem (n=60)	29 (48.3)
11. Jaw pain (N=59)	16 (27.1)
12. Loss of consciousness/fainting (N=59)	21 (35.6)
13. Nausea/vomiting (N=59)	20 (33.9)
14. Neck pain (N=58)	26 (44.8)
15. Numbness/tingling in arm or hand^‡^ N=60	26 (43.3)
16. Pale, ashen, loss/change of color (N=59)	16 (27.1)
17. Palpitations/rapid heart rate (N=59)	29 (49.2)
18. Shortness of breath/difficulty breathing (N=60)	30 (50.0)
19. Slurred speech^‡^ (N=57)	37(64.9)
20. Sweating (N=60)	18 (30.0)
21. Weakness/fatigue (N=59)	12 (20.3)

* ACS = Acute Coronary Syndrome;

† S.D. = Standard Deviation;

‡ Reverse code

As shown in Table 3, all the 60 participants answered the five items of the Attitudes subscale concerning ACS. The total mean score was 11.2 (S.D.=3.5). Most of the participants were sure they could get help for themselves (mean=2.8; S.D.=0.8) and others (mean=2.6; S.D.=0.9) if they noticed a heart attack. On the other hand, most of the participants were unsure if they could recognize the signs and symptoms of a heart attack in someone else (mean=1.8; S.D.=0.9).

**Table 3:** Descriptive statistics for Attitudes subscale items - Acute Coronary Syndrome - Response Index (ACS-Response Index). Linden, Guyana, 2020

ACS*-Response Index - Attitudes subscale	Total median score (range) 11.5 (5-18)
Items	Total mean score (S.D.)^†^ 11.2 (3.5)
22. How sure are you that you could recognize the signs and symptoms of a heart attack in someone else?	1.8 (0.9)
23. How sure are you that you could recognize the signs and symptoms of a heart attack in yourself?	2.0 (0.9)
24. How sure are you that you could tell the difference between the signs or symptoms of a heart attack and other medical problems?	2.0 (1.0)
25. How sure are you that you could get help for someone if you thought they were having a heart attack?	2.6 (0.9)
26. How sure are you that you could get help for yourself if you thought you were having a heart attack?	2.8 (0.8)

* ACS = Acute Coronary Syndrome;

† S.D. = Standard Deviation

Table 4 shows the results for the Beliefs subscale. The total mean score for this subscale was 21.3 (S.D.=4.5), and the number of patients who answered the items of the Beliefs subscale ranged from 56 to 60. The item with the highest mean score in this subscale was “I would be embarrassed to go to the hospital if I thought I was having a heart attack, but I wasn’t” (mean=3.2, S.D.=0.9). The item that showed the lowest mean score was “If I thought I was having a heart attack, I would go to the hospital right away” (mean=1.7; S.D.=1.0).

**Table 4:** Descriptive statistics for Beliefs subscale items - Acute Coronary Syndrome - Response Index (ACS-Response Index). Linden, Guyana, 2020

ACS*-Response Index - Beliefs subscale	Total median score (range) 22 (7-28)
Items	Total mean score (S.D.)^†^ 21.3 (4.5)
27. If I have chest pain that doesn’t stop after 15 minutes, I should get to the hospital as soon as possible^‡^ (N=60)	2.1 (1.0)
28. I would be embarrassed to go to the hospital if I thought I was having a heart attack, but I wasn’t (N=59)	3.2 (0.9)
29. If I thought I was having a heart attack, I would wait until I was very sure before going to the hospital (N=59)	3.2 (0.9)
30. If I thought I was having a heart attack, I would rather have someone drive me to the hospital than have an ambulance come to my home (N=59)	2.6 (1.0)
31. If I’m having chest pain and I’m not very sure if it’s a heart attack, I should go to the hospital^‡^ (N=58)	2.1 (0.9)
32. Because of the cost of medical care, I would want to be absolutely sure I was having a heart attack before going to the hospital (N=58)	1.8 (0.9)
33. If I thought I was having a heart attack, I would go to the hospital right away^‡^ (N=59)	1.7 (1.0)

* ACS = Acute Coronary Syndrome;

† S.D. = Standard Deviation,

‡ Reverse code

Table 5 shows the results of the subscales of the ACS-Response Index according to sex, age, physical activity, time since the diabetes diagnosis, body mass index, and educational level. Participants diagnosed with type 2 diabetes less than 12 months ago scored higher on the Attitude subscales than those diagnosed from 12 to 36 months (*p*=0.049). The opposite was found for the Beliefs subscale (*p*=0.049). The participants 60 years old or older scored significantly higher in the Beliefs subscale (*p*=0.001) than those younger than 60 years old (Table 5). Statistically significant differences were found when comparing the scores obtained in the Knowledge (*p*=0.008) and Attitude *(p*=0.009) subscales according to education level.

**Table 5:** Comparison of Acute Coronary Syndrome - Response Index (ACS-Response Index) subscales according to sex, age, time since diabetes diagnosis, body mass index, and educational level. Linden, Guyana, 2020

Variables	ACS*-Response Index subscales					
	Knowledge		Attitude		Beliefs	
	Median (Range)	p-value	Median (Range)	p-value	Median (Range)	p-value
*Sex*		0.656^†^		0.846^†^		0.271^†^
Female (n=39)	9.5 (4.0-15.0)		11.0 (5.0-18.0)		22.0 (7.0 28.0)	
Male (n=21)	8.00 (4.0-16.0)		12.0 (6.0 16.0)		23.0 (13.0-28.0)	
*Age (years)*		0.573^†^		0.153^†^		0.001^†^
< 60 (n=41)	8.0 (4.0-16.0)		12.0 (5.0-18.0)		21.0 (7.0-28.0)	
60+ (n=45)	9.0 (4.0-14.0)		9.0 (5.0-17.0)		23.0 (15.0-27.0)	
*PA* ^‡^		0.677^†^		0.178^†^		0.335^†^
Yes (n=9)	8.0 (4.0-16.0)		11.0 (5.0-18.0)		22.0 (7.0-28.0)	
No (n=51)	11.0 (5.0-12.0)		8.0 (6.0-15.0)		25.0 (13.0-27.0)	
*Time Diag.* ^§^		0.138^†^		0.049^†^		0.049^†^
Less than 12 months (n=27)	10.0 (4.0-16.0)		13.0 (5.0-17.0)		20.0 (13.0-28.0)	
From 12 to 36 months (n=33)	8.0 (4.0-14.0)		10.0 (5.0-18.0)		23.0 (7.0-28.0)	
BMI^||^		0.646^†^		0.405^†^		0.968^†^
Not overweight (n=6)	8.0 (5.0-1720)		8.5 (6.0-15.0)		21.5 (16.0-27.0)	
Overweight (n=44)	9.0 (4.0-16.0)		12.0 (5.0-18.0)		22.0 (7.0-28.0)	
*Educational Level*		0.008^¶^		0.009^¶^		0.109^¶^
Primary (n=25)	7.5 (4.0 - 15.0)		9.0 (5.0 - 17.0)		23.0 (13.0 - 28.0)	
Secondary (n=24)	9.0 (4.0 - 14.0)		12.0 (5.0 - 18.0)		22.0 (7.0 - 28.0)	
Trade or university (n=9)	13.5 (7.0 - 16.0)		14.0 (11.0 - 15.0)		18.0 (16.0 - 27.0)	

* ACS *=* Acute Coronary Syndrome;

† Mann-Whitney test;

‡ PA = physical activity;

§ Time Diag. = Time since diabetes diagnosis;

|| BMI = Body mass index;

¶ Kruskal-Wallis test

## Discussion

This study’s results showed a deficit of knowledge among Guyanese patients with type 2 diabetes regarding ACS symptoms. This study’s participants scored lower in the ACS Knowledge subscale than those in other studies^(8,17-18)^. Other authors report a more significant number of participants with more than 70% of correct answers in the Knowledge subscale^(8,15-16)^ compared to this study’s participants; 70% of correct answers is the cut-off point adopted by other authors to indicate a satisfactory result in this subscale^(8,13,15-16)^. Only two participants (3.3%) in this study answered more than 70% of the answers in the Knowledge subscale correctly. The difference in results may be due to the characteristics of the sample. Other studies’ participants^(8,15-16)^ may have referred to their experiences considering their diagnoses of coronary heart disease and myocardial infarction, whereas only four persons reported these comorbidities in this study. These results are in line with Leventhal’s Model, which states that mental constructions about the disease are formed by a patient’s experiences and those of other familiar people^(14)^. Another reason may be related to educational level, considering that 41% of this study’s participants had attended elementary school only, which may impose challenges for health education.

A smaller percentage of this study’s participants recognized chest pain as the most typical symptom of this syndrome. In a study conducted in Nepal^(17)^, 60% of the participants could not identify a single symptom of a heart attack, and only 20% reported chest pain as a symptom of a heart attack. Other authors^(8,13,15-16,23)^ report that chest pain was the symptom with the highest score. It is important to note that the three items with the highest score in this study are not identified as symptoms of ACS as reported by the literature^(9)^. These results may be related to the profile of this study’s participants, i.e., older adults who may often confuse pain in the sternum area with pain in the stomach.

The total mean score obtained in the Attitudes subscale was similar to that obtained by Jordanian patients^(16)^; however, it is lower than that obtained by Irish^(8)^ and Lebanese patients^(15)^. In this study, the mean score obtained in the Beliefs subscale was lower than those reported by the studies previously mentioned^(8,15-16)^. The items with the highest average score in the Attitudes subscale (“How sure are you that you could get help for someone if you thought s/he was having a heart attack?” and “How sure are you that you could get help for yourself if you thought you were having a heart attack?”) reflect the action of seeking help. However, perception regarding the need to seek help in a heart attack depends on the knowledge of symptoms. In this study, the item that reflects the ability to recognize symptoms obtained the lowest mean score: “How sure are you that you could recognize the signs and symptoms of a heart attack in someone else?” Other authors^(8,24)^ state that a higher number of participants reported they should get to a hospital as soon as possible if they had chest pain that would not stop after 15 minutes. Participants from other studies^(15-16)^ were also more positive about their ability to get help for themselves if they had a heart attack than for others.

The item in the Beliefs subscale that obtained the highest mean score, “I would be embarrassed to go to the hospital if I thought I was having a heart attack, but I wasn’t” reflects a lack of knowledge about the risk factors for ACS and its severity. In Guyana, people often resort to hot drinks, such as garlic tea or anise or ginger tea, to burp in an attempt to relieve pain. Seeking the emergency service in the face of a heart attack depends on knowledge about the symptoms and how a patient should respond^(15)^. Sociocultural aspects can also influence the interpretation and responses to ACS^(25)^. Delay in seeking treatment for acute myocardial infarction contributes to increased mortality.

This study’s results revealed that the participants with more knowledge regarding ACS symptoms and the best attitudes to respond to a heart attack also had the highest educational levels. Other studies’ findings show that inappropriate knowledge was related to lower educational levels^(13,16)^. One study using the ACS-Response Index to test the effectiveness of an application that uses simulation to interact with users reports that the factor associated with greater knowledge was having a university degree^(26)^.

Several studies^(13,27)^ report that women had more knowledge of cardiovascular diseases than men, though the opposite was found in a study addressing Jordanian patients^(16)^. No significant differences were found in this study between men’s and women’s knowledge. Similar results were found in an investigation evaluating the knowledge of workers from a Nigerian University regarding heart disease risk factors^(28)^. Additionally, a literature review focusing on the knowledge of symptoms and risk factors for heart disease reports that both women and men had a low level of knowledge about ACS^(11)^.

In this study, participants 60 years old or older scored significantly higher on the Beliefs scale than their younger counterparts. However, no statistically significant differences were found between these two groups regarding the Knowledge and Attitudes subscales. Other authors report that older adults scored lower in the Knowledge^(13)^ and Attitude subscales^(15)^ than younger patients.

In this study, 76.7% of the participants reported at least one type of physical activity, mainly walking. However, no significant differences were found in knowledge measures between patients reporting a physical activity and those who did not. The number of overweight participants or with some degree of obesity was high in this study. Obesity is recognized as a public health problem in Guyana^(5)^ and is twice more frequent among women (36.6%) than men (16.0%)^(6)^.

Georgetown and Linden are the largest cities in Guyana, associated with urbanization and, consequently, with changes in eating habits, levels of physical activity, and the use of alcohol and drugs. For this reason, cardiovascular diseases are on the rise. In addition, these lifestyle changes increase exposure to other cardiovascular risk factors (e.g., diabetes, obesity, and hypertension)^(29)^. Despite public health policies to prevent and care for chronic conditions in Guyana, the widespread belief that chronic non-communicable diseases, like diabetes and cardiovascular diseases, are consequences inherent to the aging process hinders the prevention and management of these diseases^(30)^. This cultural factor may contribute to patients’ lack of interest in disease self-management. Essential self-care behavior among people with diabetes depends on the knowledge of specific disease management, relating to healthy eating habits, being physically active, blood sugar monitoring, compliance with medications, and acquiring good problem-solving skills to cope with illness-related problems and other associated conditions.

A study reports that an educational program based on the development of empowerment principles was effective for people with type 2 diabetes^(31)^. Educational strategies empower knowledge, skills, and self-confidence, enabling people with chronic conditions to assume responsibility for managing their diseases^(31)^. Therefore, the education people with diabetes receive should focus not only on expanding their knowledge about the disease but also on cultivating a self-care attitude to prevent cardiovascular problems and decrease the time they take to seek help in cardiac emergencies.

This study’s findings show that participants reporting less than 12 months since type 2 diabetes was diagnosed obtained statistically significantly higher scores in the Attitudes subscale than those reporting between 12 and 36 months. Future studies should address this aspect, considering that the second group also scored lower in the Beliefs subscale, and diabetes is a significant risk factor for the development of coronary artery disease^(2)^.

One of this study’s limitations refers to its small sample size. However, the characteristics of the participants are representative of the Guyanese population served by the public hospitals, and the results of this study need to be explored considering the population’s cultural and economic factors that affect disease management. Another limitation concerns missing data in medical records so that some comorbidities could not be confirmed.

Nonetheless, these findings contribute to educational programs focused on improving knowledge, attitudes, and beliefs on ACS among populations from other regions, such as South America and the Caribbean. In addition, these results call the attention of health professionals and policymakers to the challenges of improving health education among patients with type 2 diabetes concerning ACS symptoms and how to respond to a heart attack.

Further studies adopting more diverse samples and including patients living in rural areas are needed. In addition, it is important to investigate the types of education provided by health care professionals to type 2 diabetes patients at all levels of health care services in Guyana, including hospitals and small services in rural areas. In addition to preventing cardiovascular disease and improving the approach to type 2 diabetes self-management, educational programs can decrease delays in seeking assistance in case of a heart attack.

Patients with type 2 diabetes receiving timely and proper education provided by nurses, doctors, and other healthcare workers become familiar with the signs and symptoms of the different complications, including those concerning ACS, and have better attitudes towards the need to seek help and the importance of self-management.

## Conclusion

This study’s findings revealed that Guyanese patients with type 2 diabetes lack knowledge regarding ACS symptoms and how to respond (Attitudes) to a heart attack appropriately, i.e., they are uncertain about the appropriate actions to take for themselves and others.

Patients with the highest educational levels (trade or university degree) obtained the highest scores on the Knowledge and Attitude subscales. Participants diagnosed with type 2 diabetes less than 12 months ago scored higher on the Attitude subscales than those diagnosed between 12 and 36 months; this group also obtained the lowest scores on the Beliefs subscale. Participants 60 years old or older scored higher on the Beliefs subscale than their younger counterparts.

These findings highlight the need for the multidisciplinary healthcare team to initiate health education at the time of the clinical diagnosis to motivate patients with type 2 diabetes to cultivate positive attitudes toward self-management and help-seeking behavior.
